# Bidirectional causal relationship between 91 blood cells and endometriosis at different sites: A Mendelian randomization study

**DOI:** 10.1097/MD.0000000000046982

**Published:** 2026-01-09

**Authors:** Shu-Ping Huang, Ze-Chao Zhang, Jing Li, Wen-Jia Ding, Liang-Ying Li, Yi-Ting Zhang, Cai-Ying Xie, Wei-Hong Li

**Affiliations:** a Department of Graduate School, Guangxi University of Chinese Medicine, Nanning, Guangxi Zhuang Autonomous Region, China; b Department of Yao College of Medicine, Guangxi University of Chinese Medicine, Nanning, Guangxi Zhuang Autonomous Region, China.

**Keywords:** bidirectional causal relationship, blood cells, endometriosis, heredity, Mendelian randomization

## Abstract

To explore the genetic associations between 91 types of blood cells (BCs) and endometriosis (EMs), providing references for the treatment of EMs. Forward Mendelian randomization (MR) analysis was conducted with BC-related single nucleotide polymorphisms (SNPs) as instrumental variables and EMs as the outcome. Conversely, reverse MR analysis was performed using EMs-related SNPs as instrumental variables and BCs as the outcome. These analyses were carried out using the TwoSampleMR package in R. The forward MR analysis revealed significant genetic correlations between EMs and 4 BCs overall (*P* < .05). Specifically, significant correlations were observed between EMs and 2 BCs in the fallopian tube, 6 BCs in the intestine, 4 BCs in the ovary, 4 BCs in the pelvic peritoneum, 2 BCs in the rectovaginal septum and vagina, 5 BCs in skin scar, 7 BCs in the uterus, 9 BCs in other unclassified locations EMs (*P* < .05). The reverse MR analysis demonstrated significant genetic correlations between EMs and 3 types of BCs (*P* < .05), while no statistically significant correlations were found for the others (*P* > .05). EMsOI showed a significant genetic correlation with 1 BC (*P < *.05). No one were observed for BCs in EMsOFT. Eight BCs in EMsOO, 5 BCs in EMsOPP, 3 BCs in EMsORSV, 3 BCs in EMsOSS, 3 BCs in EMsOU and 5 BCs in U-EMs showed genetic correlations (*P < *.05). These associations between EMs and BCs exhibit a new perspective on the bidirectional causal relationships between BCs and EMs.

## 1. Introduction

Endometriosis (EMs), manifested by pain and infertility, is a chronic inflammatory disease, associated with a large disability of daily living, causing a socio-economic diastrophic problem and burden.^[1]^ EMs appears to be one of the most common benign gynecological proliferations among premenopausal women, as it is estimated that 10 to 15% of women of reproductive age suffer from pelvic endometriosis, although its biological significance remains unclear.^[2]^ Despite the widespread occurrence of this condition, our understanding of it remains limited. Current research indicates no correlation between the severity of the disease and its symptoms, and currently, there is no blood test available for the diagnosis of endometriosis.^[3]^ Blood cells (BCs) are characterized by their ease of operation and accessibility in clinical testing, making them a routine clinical examination item.^[4]^ However, there has been no breakthrough in research on the relationship between BCs and EMs, despite the potential of BCs as targets for the diagnosis and treatment of EMs. Conventional studies have not identified a direct link between BCs and EMs. Therefore, further investigations are needed to explore whether BCs play a role in promoting or inhibiting EMs through genetic pathways, whether there are specific exposures and outcomes associated with BCs and EMs, and whether there are specific associations between BCs and EMs in different parts of the body.

In view of the above problems, Mendelian randomization (MR) provides a new analytical method to clarify the association between EMs and BCs. MR constitutes an epidemiological approach that capitalizes on genetic variants as instrumental variables (IVs) to serve as surrogates for the variables of interest, thereby facilitating the evaluation of the causal repercussions of exposures on specific outcomes.^[5]^ The inherent random assignment of single nucleotide polymorphisms (SNPs) confers resistance against confounding influences. Importantly, genetic variations remain immune to the development of subsequent outcome traits, mitigating the specter of reverse causality bias.^[6]^ In the modern era, advancements in high-throughput metabolomics have enabled the concurrent measurement of various blood biomarkers.^[7]^ The relationship between BCs and SNPs has been extensively explored in a genome-wide association study (GWAS) that included 2600 samples and investigated 91 types of BCs.^[6]^ MR Method has been used to study the relationship between different BCs and EMs, in order to clarify the specific mode of action between them, and provide a new research direction for the diagnosis and treatment of EMs. In this study, a bidirectional MR Design was used to look for potential associations between BCs and EMs.

## 2. Materials and methods

### 2.1. Description of study design

**Figure 1.**
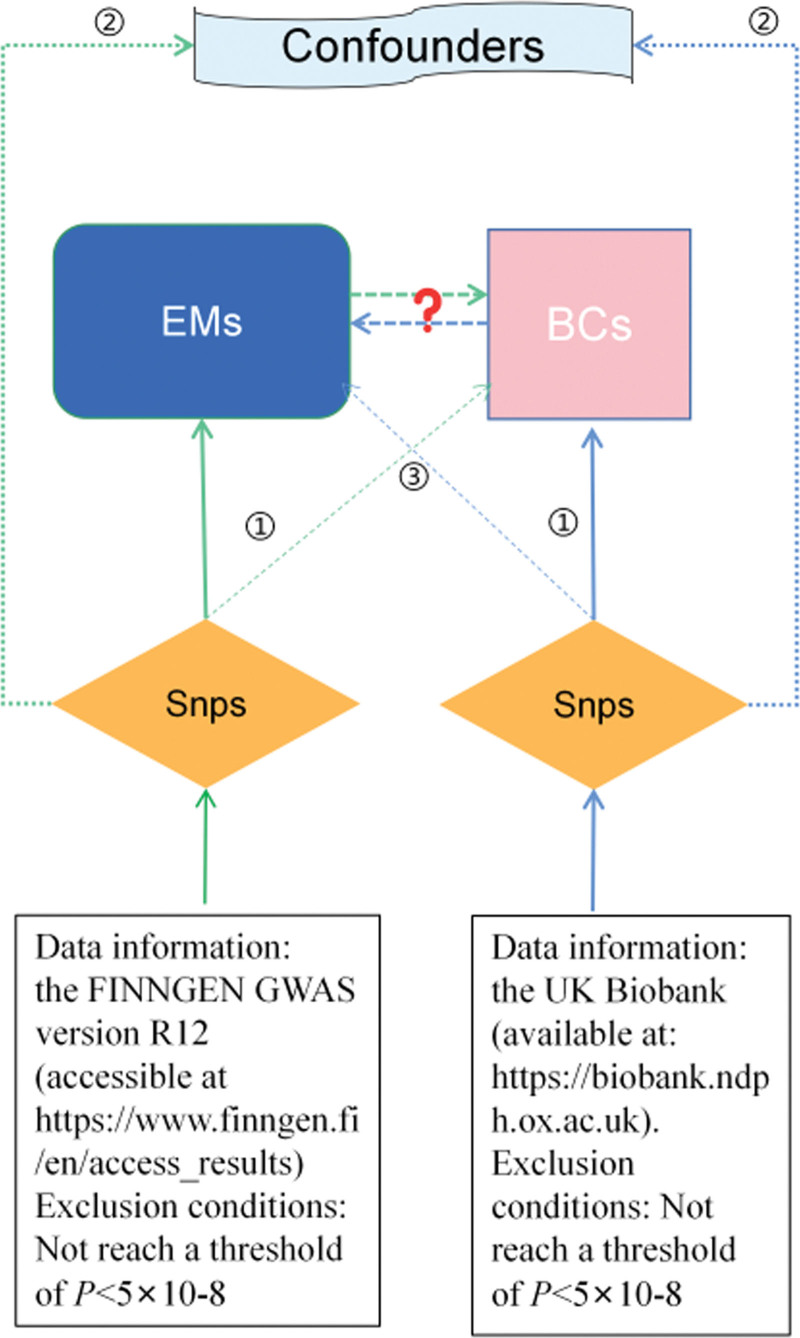
Flowchart of bidirectional MR study. The MR analysis relies on 3 core assumptions ①, ②, and ③. Assumption ① – relevance: Genes are associated with the exposure. Assumption ② – Independence: genes are not associated with any confounders of the exposure-outcome. Assumption ③ – Exclusion restriction: genes do not affect outcome expect through its potential effect on the exposure. Blue represents forward MR analysis, where BCs serve as exposures and EMs as outcomes. Green signifies reverse MR analysis, with EMs as exposures and BCs as outcomes. BCs = blood cells, EMs = endometriosis, MR = Mendelian randomization, SNP = single nucleotide polymorphism.

Figure 1 illustrates the simplified steps of this bidirectional MR study between BCs and EMs. Summary statistics from GWAS were utilized for 2 MR analyses to investigate the associations between BCs and EMs. In the forward MR analysis, BCs were set as the exposure with a selection criterion of *P* < 1 × 10^−8^, and EMs were designated as the outcome. Conversely, in the reverse MR, EMs served as the exposure with a selection criterion of *P* < 1 × 10^−8^, and BCs were the designated outcome. The core MR assumptions are outlined in Figure 1. As this study was based on publicly available databases, ethical approval was not required.

### 2.2. Selection of MR tool variables

IVs for MR analysis were chosen from 2 distinct GWAS summary results. Initially, SNPs associated with the exposure group were screened under a genome-wide significance threshold of *P* < 5 × 10^−8^.^[8]^ Secondly, the independence between the selected SNPs was evaluated based on pairwise linkage disequilibrium. SNPs associated with multiple SNPs or linked to higher *P*-values were excluded when *r*^2^ > 0.001 within a clustering window of 10,000 kb.^[9]^ Linkage disequilibrium refers to the nonrandom association between alleles at different loci. Simply put, any degree of linkage is exhibited as long as 2 genes are not inherited completely independently. Here, *r*^2^ represents a value between 0 and 1, where *r*^2^ = 1 indicates a complete linkage disequilibrium relationship between 2 SNPs, while *r*^2^ = 0 signifies complete linkage equilibrium, meaning the distribution of these 2 SNPs is entirely random. In the context of *r*^2^ = 0.001 and 10,000 kb, it implies the removal of SNPs with an *r*^2^ >0.001 within a 10,000 kb range. Thirdly, the *F*-statistic was calculated to verify the strength of individual SNPs. SNPs with an *F*-statistic >10 were considered sufficiently robust to mitigate the influence of potential biases.^[10]^

### 2.3. Data sources and instrumental variable selection for EMs

The data for EMs was sourced from the FINNGEN GWAS version R12 (accessible at https://www.finngen.fi/en/access_results). This population-based dataset primarily focuses on EMs as the main diagnosis and further includes subcategories such as Endometriosis of uterus (EMsOU), Endometriosis of intestine (EMsOI), Endometriosis of ovary (EMsOO), Endometriosis of pelvic peritoneum (EMsOPP), Endometriosis of rectovaginal septum and vagina (EMsORSV), Endometriosis of fallopian tube (EMsOFT), Endometriosis of skin scar (EMsOSS), and Unspecified/other endometriosis (U-EMs).

### 2.4. Data sources and instrumental variable selection for BCs

A summary-level GWAS dataset encompassing 91 types of BCs were acquired from the UK Biobank (available at: https://biobank.ndph.ox.ac.uk) (Table 1).

**Table 1:** Data sources.

Traits	Sample size	Year	Web source
Blood cells	2600	2023	https://biobank.ndph.ox.ac.uk/
N14_ENDOMETRIOSIS	20,190	2024	https://r12.finngen.fi/
N14_ENDOMETRIOSIS_FALLOPIAN_TUBE	280	2024	https://r12.finngen.fi/
N14_ENDOMETRIOSIS_INTESTINE	617	2024	https://r12.finngen.fi/
N14_ENDOMETRIOSIS_NOS	4152	2024	https://r12.finngen.fi/
N14_ENDOMETRIOSIS_OVARY	7878	2024	https://r12.finngen.fi/
N14_ENDOMETRIOSIS_PELVICPERITONEUM	7617	2024	https://r12.finngen.fi/
N14_ENDOMETRIOSIS_RECTPVAGSEPT_VAGINA	3226	2024	https://r12.finngen.fi/
N14_ENDOMETRIOSIS_SKIN_SCAR	118	2024	https://r12.finngen.fi/
N14_ENDOMETRIOSIS_UTERUS	5673	2024	https://r12.finngen.fi/

### 2.5. MR statistical analysis

SNPs of BCs and EMs were utilized for subsequent forward and reverse MR analyses. The inverse-variance weighted (IVW) method, grounded in all core assumptions of MR, serves as the primary statistical approach to estimate the potential bidirectional causal relationship between EMs and BCs. When multiple IVs are available, IVW emerges as the most effective analytical method, as it considers variation specificity and causal estimation heterogeneity. Additionally, sensitivity analyses, including simple mode, weighted mode, weighted median, and MR-Egger regression methods, were conducted to assess the robustness of the study findings.^[11]^ However, if IVs influence outcomes through alternative pathways, it suggests the presence of potential horizontal pleiotropic effects, and the causal estimates from IVW may be biased. Hence, MR-Egger was employed for horizontal pleiotropy testing; a *P*-value > .05 indicates the absence of horizontal pleiotropy. The MR heterogeneity test was used to ascertain heterogeneity among SNPs. If heterogeneity exists, a random-effects model was adopted; otherwise, a fixed-effects model was the default choice. Individual SNPs were sequentially excluded from the MR analysis before conducting an overall analysis, aiming to observe the impact of each SNP on the entire MR analysis results.^[12]^ The TwoSampleMR (v.0.5.6) within the R package (V.4.3.0) was utilized for primary statistical analyses and graphical representations.^[13]^ Odds ratios (ORs) and 95% confidence intervals (CIs) represent the magnitude of change in outcome risk per standard deviation increase in the exposure factor. Statistical significance was set at *P* < .05 using Bonferroni correction.^[14]^

## 3. Results

### 3.1. Forward MR results

**Figure 2.**
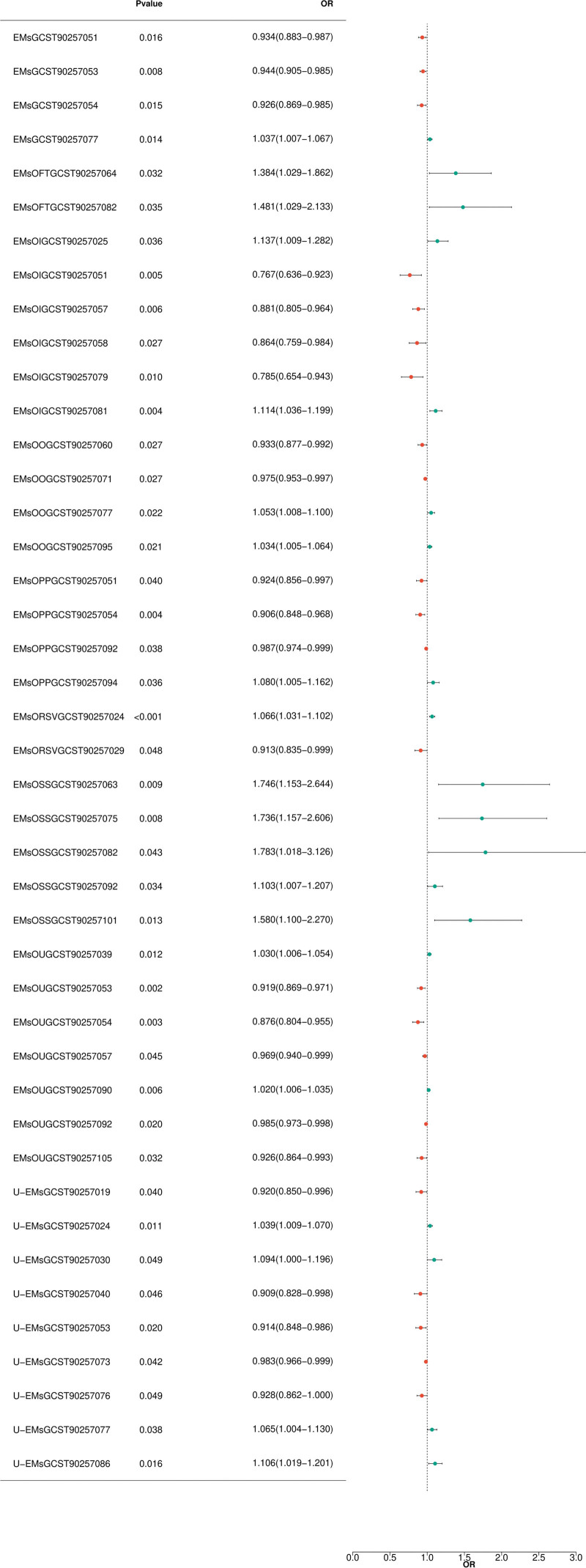
Forest plot depicting significant correlations in forward MR. The Y-axis represents diverse BCs and SNPs, while the X-axis indicates OR values. Various shapes in the graph symbolize distinct BCs ORs, with the horizontal line denoting the range of the 95% CI. The specific *P*-value and range of OR values can be seen in the figure. It can be clearly observed from the graph that different BCs and SNPs show different OR values. Some BCs have relatively high OR values, which might suggest a stronger association with the studied trait or disease. The 95% CI range plays a crucial role in interpreting the significance of these associations. Additionally, we can start to analyze the relationships between different types of BCs and SNPs based on their OR values. BCs = blood cells, CI = confidence interval, OR = odds ratio.

There is no significant horizontal pleiotropy among SNPs (Table S2, Supplemental Digital Content, https://links.lww.com/MD/R75; *P* > .05). Furthermore, by combining the Cochran *Q P*-values from the IVW and MR-Egger methods (Table S2, Supplemental Digital Content, https://links.lww.com/MD/R75; *P *> .05), no association was found to be accompanied by significant heterogeneity (Fig. 2).

#### 3.1.1. The impact of BCs on EMs

In this study, BCs were included (Table S1, Supplemental Digital Content, https://links.lww.com/MD/R75). No significant horizontal pleiotropy was observed among the SNPs (see Table S2, Supplemental Digital Content, https://links.lww.com/MD/R75; *P* > .05). Additionally, by combining the Cochran *Q P*-value from both the IVW and MR-Egger methods (see Table S2, Supplemental Digital Content, https://links.lww.com/MD/R75; *P* > .05), no associations were found to be accompanied by significant heterogeneity. With the exposure of Neutrophil perturbation response (forward scatter coefficient of variation (CV) of neutrophil-1 in response to Pam3CSK4 perturbation measured by WDF dye), the risk of EMs increases. Conversely, exposure to Neutrophil perturbation response (neutrophil-2/neutrophil-4 ratio at baseline measured by WDF dye), Neutrophil perturbation response (side fluorescence median of neutrophil-4 at baseline measured by WDF dye), Neutrophil perturbation response (side fluorescence standard deviation of neutrophil-4 at baseline measured by WDF dye), are associated with a decreased risk of EMs. Eleven SNPs were associated with an increased risk of BCs in EMs, while 40 SNPs were linked to a decreased risk of EMs. These SNPs have not been previously reported to be associated with EMs (for specific SNPs, see Table S2, Supplemental Digital Content, https://links.lww.com/MD/R75). Forward scatter (FSC) reflects cell size and volume, while the coefficient of variation (CV) indicates the heterogeneity of these parameters within a cell population.^[15]^ Pam3CSK4, a synthetic triacylated lipopeptide that mimics bacterial lipoproteins, induces an inflammatory response in neutrophils by activating the Toll-like receptor 1/2 (TLR1/2) pathway.^[16,17]^ An increase in FSC CV of neutrophils 1 after Pam3CSK4 stimulation suggests increased variability in cell size, potentially reflecting heterogeneity in their activation state or morphological changes (such as swelling or degranulation). This heterogeneity hints at a possible overreaction or dysregulation in the neutrophils’ response to inflammatory stimuli. The association of this BC with an elevated risk of EMs indicates that an excessive neutrophil response may exacerbate the chronic inflammatory microenvironment of EMs lesions.

These subpopulations may differ in maturity, activation state, or functional characteristics. Changes in the ratio may indicate shifts in neutrophil homeostasis. An increased ratio may suggest a predominance of pro-inflammatory subpopulations, while a decreased ratio may reflect a protective immune balance. The association of this BC with a reduced risk of EMs suggests that a specific distribution of neutrophil subpopulations at baseline may inhibit excessive inflammation or immune dysregulation, thereby lowering the risk of EMs.

#### 3.1.2. EMsOFT and BCs

Two BCs located in the fallopian tube region were significantly associated with EMsOFT at the genetic level (*P* < .05) (see Table S2, Supplemental Digital Content, https://links.lww.com/MD/R75). Among them, the risk of EMsOFT increased with exposure to Neutrophil perturbation response (side fluorescence standard deviation of neutrophil-4 in response to colchicine perturbation measured by WDF dye) and Monocyte perturbation response (side scatter median of monocyte 2 in response to water perturbation measured by WDF dye). 12 SNPs were associated with an increased risk of EMsOFT. The direct association of these 12 SNPs with EMsOFT has not been clearly reported and needs to be further studied (SNP information can be found in Table S2, Supplemental Digital Content, https://links.lww.com/MD/R75).

In EMs, macrophages (derived from monocyte differentiation) are highly enriched in ectopic lesions, mediating inflammatory responses and tissue remodeling.^[18]^ The enhanced response of monocytes to water perturbation may reflect their hyperactivation or dysfunctional state within the microenvironment of EMsOFT lesions. Water perturbation may simulate changes in the body’s fluid environment (such as alterations in osmotic pressure), which are common during inflammation or tissue injury.^[19]^ The association between increased SSC median and elevated EMsOFT risk suggests that monocyte responses under such stress may contribute to the survival and growth of ectopic endometrial tissue. Studies have indicated that macrophages may promote the survival of retrograde endometrial tissue and play a role in the pathophysiology of EMs.^[20]^

#### 3.1.3. EMsOI and BCs

Six BCs in the intestinal region were significantly associated with EMsOI at the genetic level (*P* < .05) (Table S2, Supplemental Digital Content, https://links.lww.com/MD/R75).

With the exposure of Red blood cell perturbation response (side fluorescence median of RBC in response to LiCl perturbation measured by platelet-F dye) and Monocyte perturbation response (side scatter median of monocyte 2 in response to TMAO perturbation measured by WDF dye), the risk of EMsOI increases. Conversely, the risk of EMsOI decreases following the exposure of Neutrophil perturbation response (neutrophil-2/neutrophil-4 ratio at baseline measured by WDF dye), Neutrophil perturbation response (side scatter CV of neutrophil-3 in response to cholic acid perturbation measured by WDF dye), Neutrophil perturbation response (side scatter standard deviation of neutrophil-4 in response to cholic acid perturbation measured by WDF dye) and Neutrophil perturbation response (forward scatter CV of neutrophil-4 in response to Pam3CSK4 perturbation measured by WDF dye). Twenty-two SNPs were associated with an increased risk of EMsOI, and 34 SNPs were associated with a decreased risk of EMsOI. These SNPs were not reported to be associated with EMsOI in the literature (details of SNPs can be found in Table S2, Supplemental Digital Content, https://links.lww.com/MD/R75).

EMsOI lesions are often accompanied by chronic inflammation and abnormal angiogenesis.^[21]^ The activation of red blood cells (RBCs) may promote ectopic endometrial tissue invasion and vascular proliferation by releasing inflammatory factors or changing the local intestinal microenvironment, thus aggravating the disease progression.

After monocytes differentiate into macrophages, they participate in immune regulation through phagocytosis, antigen presentation, and secretion of inflammatory factors.^[22]^ TMAO, a metabolite produced by intestinal flora, is associated with chronic inflammation and cardiovascular diseases.^[23,24]^ An increase in the median SSC following TMAO stimulation may indicate monocyte activation, such as an increase in lysosomes or enhanced metabolism. The metabolic level of TMAO is elevated in EMs.^[25]^ Activated monocytes may exacerbate local inflammation in the intestine, promoting immune cell infiltration and tissue fibrosis in ectopic lesions,^[26]^ thereby increasing the risk of EMsOI. Neutrophils in the inflammatory microenvironment of EMsOI may play a dual role in clearing lesions or aggravating inflammation. Specific baseline ratios can maintain immune balance and reduce EMsOI risk by limiting excessive inflammation.

Cholic acid, a bile acid with antibacterial and immunomodulatory properties,^[27]^ may induce heterogeneous neutrophil responses. Increased SSC CV suggests diverse reactivity to cholic acid. Cholic acid may modulate gut immunity and microbiota, impacting EMsOI development. Enhanced SSC CV could reflect a protective neutrophil response, reducing EMsOI risk by mitigating inflammation or microbial imbalance.

Pam3CSK4, a TLR1/2 agonist mimicking bacterial lipopeptides, triggers inflammation in endometrial epithelial cells.^[28]^ Increased FSC CV suggests heterogeneous neutrophil responses to this stimulus. Neutrophil reactions to Pam3CSK4 may regulate inflammation in EMsOI. Elevated FSC CV could indicate an adaptive response that controls inflammation, reducing EMsOI risk.

The pathogenesis of EMsOI involves chronic local inflammation in the intestine, immune imbalance, and abnormalities in the microbiota–host interaction. Six BCs suggest a dual role played by immune cells (erythrocytes, monocytes, and neutrophils) in EMsOI: a disease-promoting mechanism, where abnormal responses of erythrocytes and monocytes to LiCl/TMAO may exacerbate inflammation and oxidative stress, promoting lesion growth; and a protective mechanism, where the balance of neutrophil subpopulations and adaptive responses to cholic acid/Pam3CSK4 may inhibit disease by regulating immune homeostasis and intestinal barrier function.

#### 3.1.4. EMsOO and BCs

There are 4 BCs and EMsOO in the ovarian region that are significantly correlated at the genetic level (*P* < .05) (see Table S2, Supplemental Digital Content, https://links.lww.com/MD/R75). Among them, the risk of EMsOO increases with exposure to Neutrophil perturbation response (forward scatter CV of neutrophil-1 in response to Pam3CSK4 perturbation measured by WDF dye), Platelet perturbation response (side fluorescence standard deviation of platelet in response to isobutyric acid perturbation measured by WNR dye). Conversely, the risk of EMsOO decreases following exposure to Eosinophil perturbation response (forward scatter median of eosinophil 1 in response to colchicine perturbation measured by WDF dye), Neutrophil perturbation response (side scatter CV of neutrophil-3 in response to nigericin perturbation measured by WDF dye). 18 SNPs were associated with an increased risk of EMsOO, and 24 SNPs were associated with a decreased risk of EMsOO. For these SNPs, no association with EMsOO has been reported (see Table S2, Supplemental Digital Content, https://links.lww.com/MD/R75 for detailed SNPs).

Platelets not only participate in blood coagulation but are also crucial for the host’s immune response. They protect the vasculature from pathogens through specialized receptors, intracellular signaling cascades, and effector functions. Platelets also modulate inflammatory responses by directing innate immune cells, supporting adaptive immune surveillance, and influencing antibody production and T-cell polarization. Simultaneously, platelets contribute to tissue reconstruction and maintain vascular function following inflammatory stimulation.^[29]^ The increase in SFL SD indicates enhanced platelet heterogeneity, which may be associated with platelet activation or degranulation. The augmented intraplatelet complexity and variability under isobutyric acid perturbation could reflect a pro-inflammatory state, thereby elevating the risk of EMsOO.

Colchicine, an anti-inflammatory drug, reduces inflammatory responses by inhibiting microtubule polymerization.^[30]^ The stabilization or reduction of FSC Median may indicate controlled activation of eosinophils. Eosinophils play a crucial role in regulating allergic and inflammatory responses,^[31]^ potentially exerting a protective effect in EMsOO. The controlled reaction of eosinophils may reduce the risk of EMsOO by maintaining immune balance, decreasing excessive ovarian inflammation, and minimizing tissue damage

An increase in SSC CV suggests diverse neutrophil responses, potentially indicating an adaptive reaction. This variability may enhance neutrophils’ ability to cope with inflammatory stress. The granular variability of neutrophils under Nigericin perturbation could strengthen their protective immune function, reducing the risk of EMsOO by effectively managing inflammatory stress and minimizing chronic inflammation. These findings implicate that existing drugs, such as Nigericin, may have potential efficacy in treating EMsOO. The eosinophil and neutrophil responses induced by Colchicine and Nigericin may protect the ovaries from EMsOO by controlling inflammation and maintaining immune homeostasis.

#### 3.1.5. EMsOPP and BCs

There are 4 BCs and EMsOPP in the pelvic peritoneal region that are significantly correlated at the genetic level (*P* < .05) (see Table S2, Supplemental Digital Content, https://links.lww.com/MD/R75). Among them, the risk of EMsOPP increases with exposure to White blood cell perturbation response (side fluorescence standard deviation of WBC 2 in response to ciproflaxin perturbation measured by WNR dye). However, the risk of EMsOPP decreases after exposure to Neutrophil perturbation response (neutrophil-2/neutrophil-4 ratio at baseline measured by WDF dye), Neutrophil perturbation response (side fluorescence standard deviation of neutrophil-4 at baseline measured by WDF dye), Platelet perturbation response (forward scatter standard deviation of platelet in response to chloroform (8h) perturbation measured by WNR dye). Six SNPs were associated with an increased risk of EMsOPP, and 39 SNPs were associated with a decreased risk of EMsOPP. These SNPs have not been reported to be associated with EMsOPP (see Table S2, Supplemental Digital Content, https://links.lww.com/MD/R75 for detailed SNPs).

An increase in SFL SD suggests heightened heterogeneity in WBC 2’s response, potentially indicating an amplified inflammatory or stress reaction triggered by the antibiotic. The increased variability in WBC 2’s intracellular complexity under ciprofloxacin exposure may exacerbate the local inflammatory microenvironment. This heightened inflammation could promote the initiation and progression of EMsOPP by amplifying immune responses that favor ectopic endometrial tissue growth.

Neutrophils play a dual role in EMsOPP’s inflammatory microenvironment, potentially clearing ectopic lesions or worsening inflammation. A specific baseline Neutrophil-2/Neutrophil-4 ratio may limit excessive inflammation, thereby reducing the risk of EMsOPP by maintaining a balanced immune response that prevents disease progression.

The baseline variability in Neutrophil-4’s intracellular complexity may reduce EMsOPP risk by promoting immune equilibrium. This stability could prevent excessive inflammation and tissue damage in the pelvic peritoneum, protecting against the development of endometriotic lesions.

In EMsOPP, platelets contribute to lesion development by promoting angiogenesis and inflammation in the pelvic peritoneum. The enhanced size variability of platelets under chloroform perturbation may represent a protective response, possibly by regulating inflammation or aiding tissue repair, thus lowering EMsOPP risk.

#### 3.1.6. EMsORSV and BCs

There are 2 BCs and EMsORSVs in the rectovaginal septum and vaginal area that are significantly correlated at the genetic level (*P* < .05) (see Table S2, Supplemental Digital Content, https://links.lww.com/MD/R75). Among them, with the exposure of Platelet perturbation response (side scatter standard deviation of platelet in response to LiCl perturbation measured by platelet-F dye), the risk of EMsORSV increases. However, the risk of EMsORSV decreases after exposure to Red blood cell perturbation response (side fluorescence CV of RBC 1 in response to alhydrogel perturbation measured by reticulocyte dye). Seventeen SNPs were associated with an increased risk of EMsORSV, and 9 SNPs were associated with a decreased risk of EMsORSV; these SNPs were not reported to be associated with EMsORSV (see Table S2, Supplemental Digital Content, https://links.lww.com/MD/R75 for detailed SNPs).

This is assessed through flow cytometry, where side scatter reflects the physical properties of platelets under perturbation. An elevated SSC SD in response to LiCl may reflect a propensity for exaggerated platelet-mediated inflammation. This heightened inflammatory state could facilitate the initiation and progression of EMsORSV by creating a microenvironment that supports the survival and growth of ectopic endometrial tissue. The genetic association (*P* < .05) implies that inherited variations in this platelet response contribute to increased EMsORSV susceptibility.

Red blood cell perturbation response (side fluorescence CV of RBC 1 in response to alhydrogel perturbation measured by reticulocyte dye), the SFL CV measures the variability in intracellular complexity or fluorescence intensity of RBC 1 under alhydrogel stress, detected via flow cytometry. This reflects changes in the cellular properties of a specific RBC subgroup. Alhydrogel, an aluminum-based adjuvant, induces cellular stress responses.^[32]^ An increase in SFL CV indicates greater variability in the internal complexity of RBC 1, potentially signifying an adaptive response to stress that enhances cellular resilience or immune regulation. The increased SFL CV of RBC 1 following alhydrogel exposure is associated with a reduced risk of EMsORSV. This variability may reflect a protective mechanism that stabilizes immune responses and mitigates excessive inflammation in the rectovaginal septum and vagina. By maintaining immune homeostasis, this RBC adaptation could inhibit the pathological processes – such as tissue damage and ectopic lesion formation – that drive EMsORSV development. The significant genetic correlation (*P* < .05) suggests that inherited traits influencing this RBC response confer protection against EMsORSV.

#### 3.1.7. EMsOSS and BCs

There are 5 BCs and EMsOSS that are significantly correlated at the genetic level (*P* < .05) at skin scar sites (see Table S2, Supplemental Digital Content, https://links.lww.com/MD/R75). Among them, the risk of EMsOSS increases with exposure to Neutrophil perturbation response (side fluorescence CV of neutrophil-4 in response to colchicine perturbation measured by WDF dye), Monocyte perturbation response (side scatter median of monocyte 2 in response to Pam3CSK4 perturbation measured by WDF dye), Monocyte perturbation response (side scatter median of monocyte 2 in response to water perturbation measured by WDF dye), Platelet perturbation response (forward scatter standard deviation of platelet in response to chloroform (8h) perturbation measured by WNR dye), White blood cell perturbation response (forward scatter CV of WBC in response to Pam3CSK4 perturbation measured by WNR dye). Forty-seven SNPs were associated with an increased risk of EMsOSS, but these SNPs had not been reported to be associated with EMsOSS (see Table S2, Supplemental Digital Content, https://links.lww.com/MD/R75 for specific SNPs).

Neutrophils play a crucial role in clearing pathogens and initiating inflammatory responses during wound healing. However, their excessive activation can lead to uncontrolled inflammation, promoting the implantation of ectopic endometrial tissue. It has been demonstrated that neutrophils and the cytokines they release are present at higher levels in the circulatory system and peritoneal fluid of patients with endometriosis. This elevation contributes to the proliferation, invasion, and angiogenesis of endometrial cells.^[33]^

Monocytes participate in the inflammation and tissue remodeling of EMsOSS through their differentiation into macrophages.^[34]^ Various stress conditions may exist within EMsOSS lesions, and the abnormal response of monocytes to hydrodynamic perturbations could indicate their dysfunction in pathological environments, thereby contributing to disease progression.

Platelet perturbation response, increased platelet variability may reflect a pro-inflammatory or pro-repair state, elevating the risk of EMsOSS.

White blood cell perturbation response, leukocytes are central players in inflammatory responses, and their dysregulated activation may exacerbate the inflammatory microenvironment of EMsOSS, driving disease progression.

The occurrence of EMsOSS is associated with chronic inflammation, immune dysregulation, and abnormal tissue repair. The aforementioned BCs are all related to the responses of immune cells (including neutrophils, monocytes, platelets, and leukocytes) under specific perturbations (such as colchicine, Pam3CSK4, water, and chloroform). These perturbations may mimic inflammatory, infectious, or stress states within EMsOSS lesions. The excessive activation of neutrophils and monocytes leads to the release of pro-inflammatory factors, which sustain chronic inflammation. Monocytes differentiate into macrophages, and platelets release growth factors, promoting the survival and angiogenesis of ectopic endometrial tissue. Dysfunction of immune cells interferes with the normal healing process, increasing the likelihood of endometrial implantation.

#### 3.1.8. EMsOU and BCs

Seven BCs in the uterine region were significantly associated with EMsOU at the genetic level (*P* < .05) (see Table S2, Supplemental Digital Content, https://links.lww.com/MD/R75). Among these, the risk of EMsOU increased with exposure to Platelet perturbation response (side scatter standard deviation of platelet in response to Pam3CSK4 perturbation measured by reticulocyte dye) and Platelet perturbation response (side fluorescence CV of platelet in response to chloroform (1h) perturbation measured by WNR dye). Conversely, the risk of EMsOU decreased following exposure to Neutrophil perturbation response (side fluorescence median of neutrophil-4 at baseline measured by WDF dye), Neutrophil perturbation response (side fluorescence standard deviation of neutrophil-4 at baseline measured by WDF dye), Neutrophil perturbation response (side scatter CV of neutrophil-3 in response to cholic acid perturbation measured by WDF dye), Platelet perturbation response (forward scatter standard deviation of platelet in response to chloroform (8h) perturbation measured by WNR dye), and White blood cell perturbation response (forward scatter median of WBC 2 in response to water perturbation measured by WNR dye). Twenty-six SNPs were associated with an increased risk of EMsOU, and 66 SNPs were associated with a decreased risk of EMsOU. These SNPs were not reported to be associated with EMsOU (see Table S2, Supplemental Digital Content, https://links.lww.com/MD/R75 for specific SNPs).

Specifically, an increased proportion of platelet pro-inflammatory subpopulations may exacerbate inflammatory responses in the endometrial microenvironment, facilitating inflammatory infiltration and angiogenesis in ectopic lesions, thereby increasing the risk of EMsOU.

Abnormal activation of stressed platelets may release profibrotic factors (such as TGF-β), promoting endometrial stromal cell proliferation and fibrosis, accelerating lesion invasion, and increasing the risk of EMs and other uterine conditions.^[35]^

Neutrophil perturbation response, lateral fluorescence median (SFL median) reflect the average granularity or internal complexity of the neutrophil-4 population. Neutrophil-4 with high granular content may maintain tissue immune surveillance function in a resting state, promptly eliminating apoptotic cells or pathogens. This subpopulation suppresses abnormal inflammatory responses in the endometrial microenvironment by releasing antimicrobial peptides or anti-inflammatory mediators, thereby reducing the formation of ectopic lesions and lowering the risk of EMsOU.

This functional diversity enables the subpopulation of neutrophil to respond rapidly to local inflammatory signals while avoiding tissue damage caused by overactivation, thereby inhibiting the progression of EMsOU.

Cholic acid regulates neutrophil function through the farnesoid X receptor (FXR) or G protein-coupled bile acid receptor (TGR5), participating in inflammatory responses.^[36,37]^ An increase in granularity heterogeneity may indicate a diversified response of the 3 neutrophil subpopulations to cholic acid stimulation. This suggests that cholic acid may reduce the risk of EMsOU by decreasing neutrophil-driven pelvic inflammation.

An increase in FSC SD suggests volume heterogeneity, indicating platelet subpopulation differentiation. Adaptive differentiation may limit excessive platelet activation, reduce the release of pro-inflammatory factors, inhibit lesion angiogenesis, and thereby lower the risk of EMsOU.

White blood cell perturbation response, median forward scatter (FSC median) reflect the average size of the leukocyte subset 2. Changes in osmotic pressure may activate ion channels or stress signaling pathways. The stable size maintenance (moderate FSC median) of leukocyte subset 2 in response to water stimulation may indicate its stress resistance, suppressing the activation of inflammasomes, reducing the release of pro-inflammatory factors, and thereby protecting EMsOU.

#### 3.1.9. U-EMs and BCs

Nine BCs and U-EMs in other unclassified locations were significantly correlated at the genetic level (*P* < .05) (see Table S2, Supplemental Digital Content, https://links.lww.com/MD/R75). Among them, the risk of U-EMs increased with exposure to Platelet perturbation response (measured by the side scatter standard deviation of platelet in response to LiCl perturbation using platelet-F dye), Reticulocyte perturbation response (measured by the side fluorescence CV of reticulocyte 2 at baseline using reticulocyte dye), Neutrophil perturbation response (measured by the forward scatter CV of neutrophil-1 in response to Pam3CSK4 perturbation using WDF dye), and Monocyte perturbation response (measured by the forward scatter CV of monocyte 2 in response to water perturbation using WDF dye). Conversely, the risk of U-EMs decreased after exposure to Immature platelet fraction perturbation response (measured by the side fluorescence standard deviation of IPF in response to ciproflaxin perturbation using platelet-F dye), Red blood cell perturbation response (measured by the forward scatter standard deviation of RBC in response to rotenone perturbation using reticulocyte dye), Neutrophil perturbation response (measured by the side fluorescence median of neutrophil-4 at baseline using WDF dye), Eosinophil perturbation response (measured by the forward scatter standard deviation of eosinophil 2 in response to nigericin perturbation using WDF dye), and Monocyte perturbation response (measured by the forward scatter CV of monocyte in response to Pam3CSK4 perturbation using WDF dye). Fifty SNPs were associated with an increased risk of U-EMs, and 58 SNPs were associated with a decreased risk of U-EMs. These SNPs were not reported to be associated with U-EMs (see Table S2, Supplemental Digital Content, https://links.lww.com/MD/R75 for specific SNPs).

Platelet perturbation response, LiCl enhances Wnt/β-catenin signaling by inhibiting GSK-3β, promoting cell proliferation and inflammatory responses. An increase in platelet granularity heterogeneity may reflect subpopulation differentiation, where platelet-derived Extracellular Vesicles form aggregates with white blood cells, including neutrophils, potentially promoting inflammation. Platelet-derived chemokines, such as CXCL4 and CCL5, drive the recruitment of immune cells to plaques and facilitate inflammation.^[38]^ Activated platelets contribute to lesion angiogenesis through a pro-inflammatory microenvironment, thereby increasing the risk of U-EMs.

Reticulocyte perturbation response, suggests RNA residual in immature erythrocytes is involved in hemoglobin synthesis and oxidative stress regulation. An increase in RNA content heterogeneity may indicate bone marrow stress, leading to the release of oxidative stress mediators such as ROS. Enhanced oxidative stress could potentially damage pelvic tissues, promote ectopic endometrial adhesion and inflammatory responses,^[39]^ thereby elevating the risk of U-EMs.

Neutrophil perturbation response, Pam3CSK4 induces the release of NETs and pro-inflammatory factors from neutrophils.^[40]^ The increased heterogeneity in neutrophil size may reflect subpopulation differentiation, suggesting dysregulation of inflammatory responses. Overactivation of neutrophils may contribute to the formation of ectopic lesions through NET-mediated inflammatory cascade reactions, thereby increasing the risk of U-EMs.^[41]^

Monocyte perturbation response, water disturbance may trigger the differentiation of activated monocyte subset 2 into M1-type macrophages. These macrophages secrete pro-inflammatory factors,^[42]^ which can exacerbate pelvic inflammation and promote the progression of U-EMs.

Immature platelet fraction perturbation response, mature platelets are known to promote angiogenesis.^[43]^ A decrease in the proportion of immature platelets indicates a potential future reduction in platelet count, which may lead to a decrease in pathological angiogenesis and restrict the blood supply to ectopic lesions, thereby reducing the risk of U-EMs.

Red blood cell perturbation response, a subpopulation of RBCs with strong antioxidant capacity may scavenge ROS, thereby reducing oxidative damage to pelvic tissues. This can inhibit the growth of ectopic endometrium and lower the risk of U-EMs.

Neutrophil perturbation response, a subpopulation that can reduce the risk of U-EMs.

Eosinophil perturbation response (forward scatter standard deviation of eosinophil 2 in response to nigericin perturbation measured by WDF dye), highlights the critical role of eosinophils not only in the pathophysiology of eosinophil-related diseases but also in maintaining homeostasis, defending against infections, immune modulation through the classic Th1/Th2 balance regulation, and exerting anti-inflammatory effects.^[44]^ These profound biological functions serve to protect U-EMs.

Monocyte perturbation response, M1 macrophages play a dominant role in suppressing EM by inhibiting angiogenesis and invasive capabilities through the NF-κB pathway,^[45]^ thereby lowering the risk of U-EMs (Fig. 2).

### 3.2. Reverse MR results

**Figure 3.**
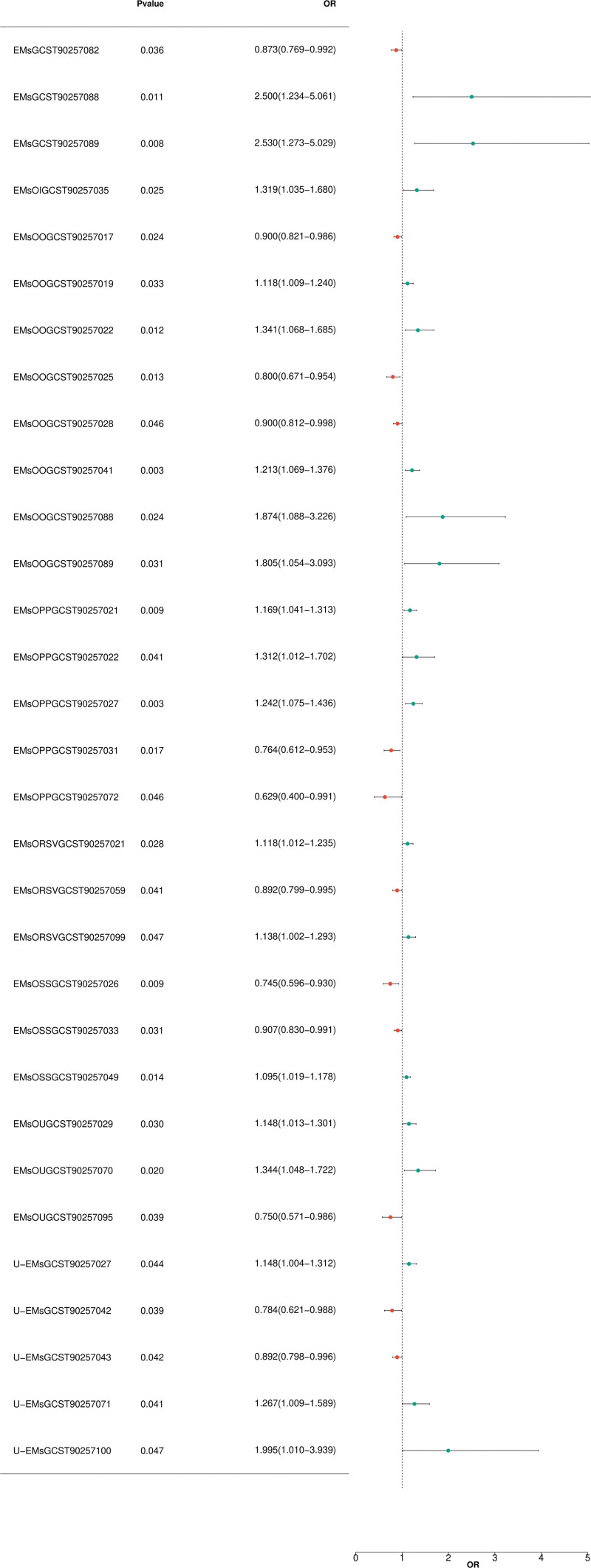
Forest plot illustrating significant correlations in reverse MR. The Y-axis represents distinct EMs and SNPs, whereas the X-axis denotes OR values. Distinct shapes within the graph symbolize distinct EMs ORs, with the horizontal line marking the range of the 95% CI. The specific *P*-value and range of OR values can be seen in the figure. It can be clearly observed from the graph that different BCs and SNPs show different OR values. Some BCs have relatively high OR values, which might suggest a stronger association with the studied trait or disease. The 95% CI range plays a crucial role in interpreting the significance of these associations. Additionally, we can start to analyze the relationships between different types of BCs and SNPs based on their OR values. BCs = blood cells, CI = confidence interval, EMs = endometriosis, MR = Mendelian randomization, OR = odds ratio, SNPs = single nucleotide polymorphisms.

There is no significant horizontal pleiotropy among SNPs (Table S2, Supplemental Digital Content, https://links.lww.com/MD/R75; *P* > .05). Furthermore, by combining the Cochran *Q P*-values from the IVW and MR-Egger methods (Table S2, Supplemental Digital Content, https://links.lww.com/MD/R75; *P *> .05), no association was found to be accompanied by significant heterogeneity (Fig. 3).

#### 3.2.1. EMs and BCs

The IVW results indicate a significant genetic association between EMs and 3 types of BCs (*P* < .05). The combined results from the forest plot reveal that with increasing exposure to EMs, there is an elevated risk for the outcomes of Platelet perturbation response (forward scatter standard deviation of platelet in response to captopril perturbation measured by WNR dye) and Platelet perturbation response (side scatter standard deviation of platelet in response to captopril perturbation measured by WNR dye), while the risk for the outcome of Monocyte perturbation response (side scatter median of monocyte 2 in response to water perturbation measured by WDF dye) decreases (Table S2, Supplemental Digital Content, https://links.lww.com/MD/R75; *P* < .05). In EMs, 200 SNPs were associated with an increased risk of exposure to 2 BCs and 103 SNPs were associated with a decreased risk of exposure to 1 BC. These SNPs were not reported in the literature related to EMs (specific SNP information can be found in Table S2, Supplemental Digital Content, https://links.lww.com/MD/R75).

#### 3.2.2. EMsOI and BCs

EMsOI is genetically significantly correlated with a type of BCs, namely Red blood cell perturbation response (measured by the forward scatter standard deviation of RBC 1 in response to DMSO perturbation using reticulocyte dye), showing a positive correlation (Table S2, Supplemental Digital Content, https://links.lww.com/MD/R75; *P* < .05). Twenty-five SNPs in EMsOI were associated with an increased risk of exposure to 1 BC, but these SNPs were not reported in the literature related to EMsOI (specific SNP information can be found in Table S2, Supplemental Digital Content, https://links.lww.com/MD/R75).

#### 3.2.3. EMsOO and BCs

Genetic correlations exist between the exposures of 8 BCs and EMs in EMsOO (*P* < .05). Specifically, with EMsOO exposure, there is an increased risk of Immature platelet fraction perturbation response (measured by the side fluorescence standard deviation of IPF in response to ciprofloxacin perturbation using platelet-F dye), Immature platelet fraction perturbation response (measured by the forward scatter standard deviation of IPF in response to isobutyric acid perturbation using platelet-F dye), Reticulocyte perturbation response (measured by the side fluorescence CV of reticulocyte 1 in response to rotenone perturbation using reticulocyte dye), Platelet perturbation response (measured by the forward scatter standard deviation of platelet in response to captopril perturbation using WNR dye), and Platelet perturbation response (measured by the side scatter standard deviation of platelet in response to captopril perturbation using WNR dye). Conversely, there is a decreased risk of White blood cell perturbation response (measured by the SSC median of WBC 1 at baseline using platelet-F dye), Red blood cell perturbation response (measured by the side fluorescence median of RBC in response to LiCl perturbation using platelet-F dye), and Red blood cell perturbation response (measured by the RBC percentage in response to Pam3CSK4 perturbation using platelet-F dye). In EMsOO, 377 SNPs were associated with an increased risk of exposure to 5 BCs and 228 SNPs were associated with a decreased risk of exposure to 3 BCs. These SNPs were not reported in the literature related to EMsOO (detailed SNP information can be found in Table S2, Supplemental Digital Content, https://links.lww.com/MD/R75).

#### 3.2.4. EMsOFT and BCs

EMsOFT, as a genetic exposure factor, did not show any statistically significant genetic correlation with BCs.

#### 3.2.5. EMsOPP and BCs

Genetic correlations exist among the 5 BCs in EMsOPP (*P* < .05). With increasing exposure to EMsOPP, the risks of the following responses increase: Red blood cell perturbation response (measured by the side fluorescence CV of RBC in response to H2O2 perturbation using platelet-F dye), Immature platelet fraction perturbation response (measured by the forward scatter standard deviation of IPF in response to isobutyric acid perturbation using platelet-F dye), and another Red blood cell perturbation response (measured by the forward scatter standard deviation of RBC in response to nigericin perturbation using platelet-F dye). Conversely, the risks of Reticulocyte perturbation response (measured by reticulocyte 2 count in response to captopril perturbation using reticulocyte dye) and Eosinophil perturbation response (measured by the side scatter CV of eosinophil 2 in response to nigericin perturbation using WDF dye) decrease. In EMsOPP, 187 SNPs were associated with an increased risk of exposure to 3 kinds of BCs, and 121 SNPs were associated with a decreased risk of exposure to 2 kinds of BCs. These SNPs were not reported in the literature related to EMsOPP (specific SNP information can be found in Table S2, Supplemental Digital Content, https://links.lww.com/MD/R75).

#### 3.2.6. EMsORSV and BCs

Genetic correlations exist among the 3 BCs in EMsORSV (*P* < .05). Specifically, with exposure to EMsORSV, the risks of Red blood cell perturbation response (measured by the side fluorescence CV of RBC in response to H2O2 perturbation using platelet-F dye) and White blood cell perturbation response (measured by the side scatter CV of WBC 2 in response to nigericin perturbation using WNR dye) increase, while the risk of Monocyte perturbation response (measured by the side fluorescence standard deviation of monocyte 2 in response to ciprofloxacin perturbation using WDF dye) decreases. 79 SNPs in EMsORSV were associated with an increased risk of exposure to 2 BCs and 39 SNPs were associated with a decreased risk of exposure to 1 BC; these SNPs were not reported in the literature related to EMsORSV (detailed SNP information is available in Table S2, Supplemental Digital Content, https://links.lww.com/MD/R75).

#### 3.2.7. EMsOSS and BCs

Genetic correlations exist among the 3 BCs in EMsOSS (*P* < .05). Specifically, with exposure to EMsOSS, there is an increased risk of Neutrophil perturbation response (measured by the forward scatter median of neutrophil-1 at baseline using WDF dye). Conversely, the risks of Red blood cell perturbation response (measured by the side fluorescence standard deviation of RBC in response to nigericin perturbation using platelet-F dye) and Platelet perturbation response (measured by the side fluorescence CV of platelet in response to colchicine perturbation using reticulocyte dye) decrease. In the EMsOSS, 4 SNPs were associated with an increased risk of exposure to 1 BC, and 7 SNPs were associated with a decreased risk of exposure to 2 BCs. These SNPs were not reported in the literature related to EMsOSS (specific SNP information can be found in Table S2, Supplemental Digital Content, https://links.lww.com/MD/R75).

#### 3.2.8. EMsOU and BCs

Genetic correlations exist among the 3 BCs in EMsOU (*P* < .05). Specifically, with the exposure of EMsOU, there is an increased risk of Red blood cell perturbation response (measured by the side fluorescence CV of RBC 1 in response to alhydrogel perturbation using reticulocyte dye) and Neutrophil perturbation response (measured by the forward scatter CV of neutrophil-1 in response to LiCl perturbation using WDF dye). Conversely, the risk of Platelet perturbation response (measured by the side fluorescence standard deviation of platelet in response to isobutyric acid perturbation using WNR dye) decreases. In EMsOU, 87 SNPs were associated with an increased risk of exposure to 2 BCs, and 42 SNPs were associated with a decreased risk of exposure to 1 BC. These SNPs were not reported in the literature related to EMsOU (specific SNP information can be found in Table S2, Supplemental Digital Content, https://links.lww.com/MD/R75).

#### 3.2.9. U-EMs and BCs

Genetic correlations exist among the 5 BCs in U-EMs (*P* < .05). Specifically, with the exposure to U-EMs, the risks of Red blood cell perturbation response (measured by the forward scatter standard deviation of RBC in response to nigericin perturbation using platelet-F dye), Neutrophil perturbation response (measured by the side scatter CV of neutrophil-3 in response to nigericin perturbation using WDF dye), and Unknown cell population perturbation response (measured by the forward scatter standard deviation of unknown cell population 1 in response to nigericin perturbation using WNR dye) increase. Conversely, the risks of Red blood cell perturbation response (measured by the side fluorescence median of RBC 2 in response to TMAO perturbation using reticulocyte dye) and Neutrophil perturbation response (measured by the side fluorescence standard deviation of neutrophil-4 in response to alhydrogel perturbation using WDF dye) decrease. In U-EMs, 133 SNPs were associated with increased risk of exposure to 3 kinds of BCs, and 88 SNPs were associated with reduced risk of exposure to 2 kinds of BCs. These SNPs were not reported in the literature related to U-EMs (specific SNP information can be found in Table S2, Supplemental Digital Content, https://links.lww.com/MD/R75).

Differences in various BCs exist among EMs located in different body parts, and some of these differences have been investigated in relevant studies to elucidate their mechanisms. Chronic inflammation persistently present in the bodies of patients with EMs leads to enhanced platelet activation, promoting fibrosis within the EMs.^[46]^ EMsThe chronic inflammation in EMs activates the JAK2/STAT3 pathway,^[47]^ which stimulates megakaryocyte proliferation and the release of platelet precursors.^[48,49]^ The hypoxic microenvironment of EMs lesions may induce autophagy, reducing the complexity of cellular contents.^[50]^ Patients with EMs experience an increase in ROS,^[51]^ which causes the disintegration of red blood cell membranes and potential microparticle formation, resulting in fragile red blood cell membranes.^[52]^ Persistent inflammatory signals in EMs can lead to changes in leukocyte subpopulations.^[53]^ Erythropoietin plays a certain role in promoting the initial stages of peritoneal EMs.^[54]^ However, some of the conclusions revealed in this study still require further confirmation.

## 4. Discussion

In this 2-way MR study, we discovered a genetic association between EMs and BCs, excluding heterogeneity and pleiotropy. The varying levels of increase and decrease in BCs among EMs in different locations provide a foundation for further research and can serve as a diagnostic indicator for EMs in specific areas. That is, when a particular BC is found to be elevated or decreased, it may be an external manifestation of EMs in a specific location. The differences in BCs among EMs in various parts also offer research value for the prognosis and treatment of EMs in distinct regions. On one hand, this may explain the varying severity of EMs in different locations, and on the other hand, these BCs may become targeted therapeutic points for treating EMs in specific positions in the future.

This untangles the functional differentiation of neutrophils in EMs across various environments and locations. Monocytes’ granularity (SSC Median) in response to water perturbation is associated with increased risk in EMsOFT, yet it decreases risk upon EMs exposure. This could stem from monocyte activation driving inflammation in EMsOFT, whereas the long-term inflammatory state in EMs patients may deplete monocyte granule contents, leading to an inverse association.^[55]^ Erythrocytes’ response to LiCl perturbation shows an elevated risk in EMsOI, but in EMsOO exposure, the SFL median of erythrocytes to LiCl decreases. The mechanism might involve abnormally enhanced Wnt/β-catenin signaling in intestinal lesions stressing erythrocytes,^[56]^ while ovarian EMs could interfere with LiCl effects by inhibiting SFRP4, resulting in opposite outcomes. Regarding platelets’ heterogeneity in response to chloroform perturbation, an increase in platelet FSC SD after 8-hour chloroform perturbation in EMsOPP is associated with reduced risk (OR < 1). However, under the same chloroform perturbation in EMsOSS (skin scar), an elevation in platelet FSC SD increases risk (OR > 1). The contrasting effects of the same BC in different anatomical sites could be due to platelet heterogeneity possibly inhibiting excessive repair^[57]^ in the pelvic peritoneum, whereas abnormal platelet activation in skin wounds promotes fibrosis,^[58]^ reflecting differential regulation of platelet function by the microenvironment. This study clearly identifies cases of specific and inversely associated conclusions among different subtypes, primarily stemming from the heterogeneity of anatomical microenvironments, cellular subpopulation functions, and disease-immune feedback pathways. These contradictions highlight the complex pathological mechanisms of EMs and emphasize the need for future research to integrate single-cell sequencing and spatial omics to disentangle the immune regulatory networks specific to the microenvironment.

The results of this study untangle the activation of TLR1/2 (Pam3CSK4) and NLRP3 pathways, which drive the release of neutrophil NETs and macrophage polarization,^[59]^ promoting chronic inflammation in EMs lesions. This discovery offers a new perspective for understanding the pathophysiology of EMs. The release of neutrophil NETs not only participates in the regulation of inflammation^[59]^ but also potentially exerts a profound influence on the microenvironment of EMs lesions. Similarly, the polarization state of macrophages plays a pivotal role in the development of chronic inflammation, further modulating the immune response within lesions through the secretion of various cytokines and chemokines.^[60]^ Additionally, the activation of TLR1/2 and NLRP3 pathways, as upstream events in this process, may directly contribute to the persistence and exacerbation of chronic inflammation in EMs lesions.^[61]^ Future research targeting these pathways and cellular processes is expected to provide novel strategies and targets for the treatment of EMs.

The granularization maturity of neutrophil subpopulations and the stress response capability of monocyte subpopulations determine the transformation of pro-inflammatory/anti-inflammatory phenotypes,^[62]^ leading to an imbalance of immune cell subpopulations. Changes in the distribution of neutrophil subpopulations reflect differences in their maturity, activation status, or functional characteristics. The distribution of specific subpopulations may influence the intensity and direction of inflammatory responses.^[63]^ Studies have indicated that dynamic variations in neutrophil subpopulations play a significant role in immune balance, where the dominance of pro-inflammatory subpopulations may exacerbate inflammation, while the predominance of anti-inflammatory subpopulations aids in maintaining immune homeostasis.^[64]^ This imbalance may constitute one of the crucial factors contributing to the persistence and exacerbation of chronic inflammation within EMs lesions. Further investigations can delve into the specific mechanistic roles of distinct immune cell subpopulations in the pathogenesis of EMs, as well as their interactive relationships. Modulating the functions and balance of these immune cell subpopulations may offer novel insights and approaches for the treatment of EMs. Additionally, future research can focus on precise regulation of these immune cell subpopulations to achieve targeted therapy for EMs lesions, thereby enhancing therapeutic efficacy and reducing side effects.

Erythrocyte membrane lipid peroxidation (H2O2), platelet mitochondrial dysfunction (rotenone), and gut microbiota metabolites (TMAO, isobutyric acid) exacerbate tissue damage through ROS-antioxidant system imbalances.^[65,66]^ The interplay of these factors forms a complex network that not only promotes the occurrence and progression of EMs but also potentially affects treatment outcomes. For instance, H2O2, as a product of erythrocyte membrane lipid peroxidation, can induce oxidative stress and further activate inflammatory responses, thereby intensifying tissue injury.^[67]^ Platelet mitochondrial dysfunction may lead to abnormal energy metabolism, affecting the normal functions of platelets and subsequently promoting inflammatory reactions and tissue damage.^[68]^ Furthermore, gut microbiota metabolites such as TMAO and isobutyric acid, by influencing the intestinal microecological balance, may also indirectly participate in the pathogenesis of EMs.^[69,70]^ In the future, in-depth exploration of the interactions among these factors and how they collectively impact the pathogenesis and treatment of EMs will provide a more comprehensive perspective and strategies for EMs therapy.

Enhanced genetic susceptibility in EMs augments platelet morphological heterogeneity in response to captopril (manifested by increased FSC/SSC SD) and suppresses monocyte granular secretion upon water perturbation (indicated by decreased SSC median), suggesting that the disease itself remodels peripheral immune cell phenotypes through chronic inflammation. Additionally, we have identified subtype-specific inverse associations within EMs. The proportions of neutrophil subpopulations (neutrophil-2/4), platelet heterogeneity (FSC SD upon chloroform perturbation), and monocyte granularity (SSC median upon water perturbation) emerge as potential markers for EMs classification and prognosis.

Variations in these markers not only reflect abnormalities in the immune system of EMs patients but also correlate closely with disease progression and severity. As a critical component of innate immunity, changes in the proportions of neutrophil subpopulations may unveil differences in immune responses among various EMs subtypes. The elevated platelet heterogeneity, especially the increased FSC SD under chloroform perturbation, might indicate enhanced platelet activation and dysfunction, further supporting the significant role of platelets in the pathophysiology of EMs. Moreover, the reduced monocyte granularity, represented by a decrease in SSC median upon water perturbation, could suggest impaired monocyte function or maturation defects, aligning with the chronic inflammatory state observed in EMs patients. Consequently, the combined detection of these markers may offer new insights and bases for precise classification, prognosis evaluation, and individualized treatment of EMs. Targeting TLR1/2 (with Pam3CSK4 antagonist), NLRP3 (using Nigericin analogues), and bile acid receptors (via TGR5 agonists) may modulate immune cell functions, while colchicine-induced eosinophil stabilization could provide an interventional strategy for ovarian EMs.

This study still has limitations. Based on GWAS data from the European population, it remains to be validated for cross-ethnic generalizability. Most associations rely on bioinformatics predictions, requiring experimental models to verify the functions of neutrophil subpopulations and the roles of metabolic pathways. The pathological heterogeneity of anatomical subtypes may obscure specific microenvironmental mechanisms, necessitating further analysis through a combination of clinical and basic experiments. In this study, the influence of single SNP such as gender was excluded by the research method of SNP elimination one by one, and the original conclusions were still maintained (see OA_Supplemental Digital Content for details, Supplemental Digital Content, http://links.lww.com/MD/R145). Compared to traditional observational studies, the significant advantage of this research is that the results obtained through MR avoid reverse causality and confounding bias. Similarly, the comprehensive and extensive GWAS data utilized for MR analysis can enhance the accuracy of the findings. More detailed experiments are needed for validation, and we hope to elucidate the specific mechanism of action of BCs on EMs.

## 5. Conclusion

Our study, through MR analysis, has untangled the genetic associations between 91 types of BCs and EMs and its subtypes, validating the existence of bidirectional causal relationships. Abnormal neutrophil function stands as a central driver in the pathophysiology of EMs, with its activation status, subpopulation distribution, and granular characteristics regulating disease risk via inflammatory pathways. Furthermore, metabolic stress and immune response heterogeneity among monocytes, platelets, and RBCs exhibit specific associations across different anatomical subtypes of EMs.

In EMsOFT, increased granular heterogeneity of neutrophils in response to colchicine (SFL SD) and granularity of monocytes to water perturbation (SSC median) elevate risk, indicating hyperactivation of immune cells within the local inflammatory microenvironment. For EMsOI, elevated granularity of RBCs to LiCl (SFL median) and monocytes to TMAO (SSC median) exacerbate risk, while a reduced neutrophil subpopulation ratio (neutrophil-2/4) and response to cholic acid (SSC CV/SD) lower risk, suggesting a key role for the imbalance in gut microbiota-immune interactions.

In EMsOO, increased heterogeneity of platelets to isobutyric acid (SFL SD) and volume variability of neutrophils to Pam3CSK4 (FSC CV) heighten risk, whereas decreased volume stability of eosinophils to colchicine (FSC median) lowers risk, reflecting an inflammation-repair imbalance in the ovarian microenvironment. For EMsOPP, increased heterogeneity of leukocytes to ciprofloxacin (SFL SD) raises risk, while a reduced baseline neutrophil subpopulation ratio (neutrophil-2/4) and volume heterogeneity of platelets to chloroform (FSC SD) lower risk, highlighting the importance of peritoneal immune homeostasis.

Our research not only deepens the understanding of the complex pathophysiological mechanisms of EMs but also emphasizes the critical role of cellular functional diversity across different EMs subtypes. Through detailed cellular phenotyping, we have revealed associations between specific cell subpopulations and their functional characteristics with EMs risk, providing a theoretical foundation for the development of precision treatment strategies targeting specific EMs subtypes. Future studies will further explore the potential of these cellular characteristics as biomarkers and how they respond to different therapeutic interventions, aiming to achieve early diagnosis and effective management of EMs. Additionally, this study underscores the importance of investigating immune-metabolic interactions at multiple tissue levels to understand the pathophysiology of complex chronic diseases, offering new perspectives and methodologies for the study of other similar conditions.

## Author contributions

**Conceptualization:** Ze-Chao Zhang.

**Data curation:** Yi-Ting Zhang, Cai-Ying Xie.

**Formal analysis:** Yi-Ting Zhang, Cai-Ying Xie.

**Funding acquisition:** Wei-Hong Li.

**Investigation:** Shu-Ping Huang, Jing Li.

**Methodology:** Ze-Chao Zhang, Wen-Jia Ding, Liang-Ying Li.

**Software:** Jing Li, Wen-Jia Ding, Liang-Ying Li.

**Supervision:** Liang-Ying Li, Cai-Ying Xie.

**Validation:** Jing Li.

**Writing – original draft:** Shu-Ping Huang, Ze-Chao Zhang.

**Writing – review & editing:** Wei-Hong Li.

## Supplementary Material




